# Developing a strategic guideline to design an intelligence instrument applicable to South African school learners

**DOI:** 10.3389/fpsyg.2026.1738328

**Published:** 2026-02-10

**Authors:** Ilze van der Merwe, Werner de Klerk, Petro Erasmus

**Affiliations:** School of Psychosocial Health, Community Psychosocial Research (COMPRES), North-West University, Potchefstroom, South Africa

**Keywords:** cross-cultural, fair assessment, intelligence assessment, multi-ethnic equalization, school learners, South Africa, strategic guideline, valid testing

## Abstract

In addition to difficulties that arise when attempting to conduct culturally fair and valid assessment practices to multi-ethnic school learners [who vary in degree of acculturation, education, and socio-economic status (SES) backgrounds], South African practitioners also face the reality of having a shortage of up-to-date, culturally appropriate intelligence instruments. This qualitative document analysis aimed to develop a strategic guideline to design an intelligence instrument that is suitable for South African school learners of a multi-ethnic population and from multidiverse contexts. Applying a reflexive thematic approach, we analyzed two documents—articles produced during phases 1 and 2 of an overarching three‑phased PhD study—to reveal the following themes: (i) utilized intelligence measurements in current South African school learner context are less relevant; (ii) it does not seem feasible to design or adapt suitable intelligence measures that are valid and reliable in the current South African school learner context; (iii) the South African education system is a major issue specifically within lower socio-economic status contexts; and (iv) current South African school learner contextual and demographic influences need to be taken into consideration. From these themes, steps were developed that comprise the proposed strategic framework to design applicable intelligence instruments for South African school learners. The steps include both guidance towards instrument design as well as the manner and context of its application, as these features function as interrelated entities.

## Introduction

Referred to as the rainbow nation (as coined by Archbishop Desmond Tutu), South Africa (ZA) is characterised by its multi-diversity in cultures, languages, socio-economic stance (SES) environments, and scholastic systems (Sidanius et al., 2019). Attempting to administer constructive, applicable and fair intelligence assessments to scholastic learners in ZA is challenging, not only because of the diversity in its population compositions and contexts, but also the vast socio-economical, demographic, and educational changes it has undergone over the past time, with trends of acculturation towards a Westernised lifestyle (Cockcroft and Sumaya, 2022; Cockcroft et al., 2015; Shuttleworth-Edwards, 2023). Variability in degree of acculturation has caused significant variations in intelligence measured performances, where South African school learners differ essentially in terms of their language use, test-taking sophistication, level and quality of education, SES backgrounds, and contexts at home and school (Cockcroft et al., 2015; Shuttleworth-Edwards, 2023; Shuttleworth-Edwards et al., 2013). This challenge is worsened by the reality of having a shortage in neuropsychological tests that are suitable and fair to all South African school learners of a multidiverse population (Cockcroft and Sumaya, 2022).

### Historic transformation of school learner populations and contexts in ZA

During the apartheid era (1948–1994), socio-economical and educational segregation was prevalent among the four officially classified racial groups (African, Colored, Caucasian, and Indian/Asian), where the Caucasian population group was favored and predominantly living within higher SES contexts and receiving higher quality of education in well-resourced, privileged schools (Shuttleworth-Edwards, 2023; Sidanius et al., 2019; Te Nijenhuis et al., 2011). Today, after the democratization of ZA in 1994 and launching of government-induced upliftment efforts, many non-Caucasian school learners have since advanced to more privileged SES living circumstances and attend well-resourced schools that provide higher quality of education; this has consequently led to higher performances in assessments academically and intellectually, with scores almost equal to their Western, Caucasian peers (Shuttleworth-Edwards, 2023). Similar findings were seen after a government initiative to uplift Afrikaans-speaking South Africans during the apartheid era, where these school learners were placed in schools that provided higher quality of education, which yielded higher academic and intellectual performances, equivalent to their English-speaking peers (Te Nijenhuis et al., 2011). The above-mentioned findings indicate a predominant link between quality of education received at schools (within higher SES environments) and higher intelligence measured performances achieved by school learners in ZA, despite their ethnicity (Shuttleworth-Edwards, 2023; Te Nijenhuis et al., 2011).

### Historic transformation of the psychometric test industry in ZA

In the new democratic, post-apartheid ZA, legislation was passed to protect all (including those with special needs or having any contextual disadvantage) from unfair practices such as discrimination, stereotyping, or exclusion [Department of Education (DoE, South Africa), 2001; Donald et al., 1997]. New democratic legislation had enforced and motivated psychologists to undergo major paradigm shifts in their assessment practices on South African school learners (Foxcroft et al., 2004; Swart and Pettipher, 2005). Drawing attention to two pieces of legislation in particular: (i) the Education White Paper 6 in 2001 (introducing inclusive education and training), and (ii) the Employment Equity Act 55, section 8, in 1998 (enforcing non-discriminatory, fair and unbiased psychological assessments), required psychologists to move from medical deficit practices (i.e., labelling, segregation and fault-finding) to practices based on a social systems change approach of adjusting the social environment (i.e., the psychological assessment practices, findings and referrals) to fit the needs of the school learners, and not vice versa (Swart and Pettipher, 2005).

The Human Sciences Research Council (HSRC) served as the largest developer and distributer of psychometric tests during the apartheid era until it was restructured during the early 1990s, with significant changes in its research aims such as discontinuing their role of developing, revising, and adapting intelligence tests (Chisholm and Morrow, 2007; Foxcroft et al., 2004). Various smaller companies and university research teams have taken over the role of research in psychometric testing (test development, standardisation, adaptation, and review), but on a much smaller scale [see examples of such research studies in De Beer (2005), Laher and Cockcroft (2013), and Shuttleworth-Edwards and Truter (2023)]. Laher and Cockcroft (2014) acknowledge and note how the above-mentioned research efforts have helped to enhance the field of psychometric assessment; however, note the lingering challenges of shortages in skilled experts, financial resources, and constructive leadership in the field of test development and adaptation for a diverse population such as ZA. Shortages of skilled professionals to develop, adapt, and administrate assessments to culturally and linguistically diverse populations seem to be a challenging reality worldwide (Dutt et al., 2022).

### Intellectual measurement of school learners in ZA today

Considering the applicability of Western-developed intelligence measures, which are currently administered to school learners in ZA, two distinct groups can be identified, namely the higher SES, Westernised, privileged schooled (higher quality of education received), and the lower SES, non-Westernised, less privileged schooled (lower quality of education received) group of school learners, who vary in demographics (culture and language) within these two groups (Shuttleworth-Edwards, 2023). Ethically appropriate and valid cognitive testing obligates acknowledging the influencing contextual elements (namely culture, language, SES, and quality of education) of South African testees and adapting measurements and assessment practices accordingly (Cockcroft and Sumaya, 2022). Ensuring fair and applicable intelligence measurement of South African learners is a matter of ethical obligation (Cockcroft and Sumaya, 2022; Laher and Cockcroft, 2017) which, despite being challenging, should be investigated to help continue the imperative movement initiated and driven by the post-apartheid, democratic South African government.

## Problem statement

In the face of limited resources and multi-ethnic school learners who vary in degree of acculturation, education, and SES backgrounds, South African practitioners are politically and ethically obligated to administer culturally fair assessments, using valid and reliable test instruments (Cockcroft and Sumaya, 2022; Nel et al., 2016; Shuttleworth-Edwards, 2023). Intellectual measurements are applied as part of a full assessment process to determine diagnoses and intervention strategies when school learners present with challenges such as learning difficulties (Nel et al., 2016; Shuttleworth-Edwards, 2016). If performances from these measurements do not correlate with the school learner’s true performance, then inaccurate findings and subsequent intervention plans will be executed, which will be to the detriment of the child. Therefore, a need exists to develop a guideline that would suggest how to design appropriate intellectual tests and practices that would yield true findings of multi-ethnic school learners in ZA.

### Aim of the research study

The aim of this qualitative document analysis was to develop a strategic guideline to design an intelligence instrument applicable to South African school learners. This was accomplished by following a multimethod approach of merging the findings from literature themes (by means of a critical review; see Van der Merwe et al., 2022) and findings from interview themes (employing a qualitative interpretive description design; see Van der Merwe et al., 2024) to develop a strategic guideline. The qualitative document analysis was steered by the following research question: *Which elements should be incorporated into a strategic guideline to design an intelligence instrument applicable to South African school learners?*

## Method of investigation

### Research design

A qualitative document analysis (Bowen, 2009; Morgan, 2022) was conducted to develop the strategic guideline. “Trustworthy … guidelines should provide recommendations, document the development process, and highlight implementation information” (Nieuwlaat et al., 2021, p. 4721). This qualitative document analysis has received ethical approval from the Health Research Ethics Committee (HREC) of the North-West University (NWU-00191-21-A1).

### Data collection

Steered by the aim of this qualitative document analysis, data were retrieved from two studies developed in Phase 1 (Document 1) and Phase 2 (Document 2) of a PhD Research Study (Van der Merwe, 2024). Document 1 and Document 2 were published (see Van der Merwe et al., 2022, 2024). Both research studies implied a need for intelligence instruments that are suitable to South African school learners. These studies were intentionally employed as they form part of the process of developing a strategic guideline which acts as an advisory statement for more applicable and therefore sound psychological intelligence assessment instruments applied to South African school learners.

*Document 1* (Van der Merwe et al., 2022). This critical review research study aimed to search, critically appraise, and analyse scientific literature regarding intelligence instruments applied to South African school learners. The search initially yielded 405 studies, of which 15 (see August, 2017; Blake, 2011; Cassoojee, 2020; De Beer, 2005; Ferrett, 2011; Jansen and Greenop, 2008; Jinabhai et al., 2004; Levert and Jansen, 2001; Mawila, 2012; Mitchell et al., 2018; Naicker, 2013; Reid et al., 2002; Shuttleworth-Edwards et al., 2013; Teixeira, 2011; Van Wyhe, 2012) were finally included for thematic analysis. Three main themes emerged from the included studies, namely: applicability of intelligence instruments administered to South African school learners; Contextual and demographic influences affecting performance on administered intelligence instruments; and Intellectual measuring instruments related to developmental and cognitive ability levels.

*Document 2* (Van der Merwe et al., 2024). The aim of this qualitative interpretive description research study was to investigate the experiences of both experts in intelligence test development/adaptation and psychologists/psychometrists who have administered intelligence tests to South African school learners in various contexts. Twelve psychologists/psychometrists were interviewed, of which six were also experts in test development/adaptation. Findings from reflexive thematic analysis uncovered four themes: (i) utilized intelligence measurements in the current South African school learner context are less relevant; (ii) South African education system is a major issue specifically within lower SES contexts; (iii) it does not seem feasible to design or adapt suitable intelligence measures that are valid and reliable in the current South African school learner context; and (iv) key informants’ recommendations from their experiences.

### Data analysis

**Figure 1:**
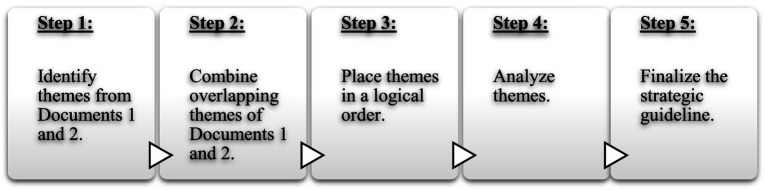
Five-step procedure for qualitative document analysis.

In this qualitative document analysis, reflexive thematic analysis, as described by Braun and Clarke (2021) and Byrne (2022), was applied whilst pursuing the following five steps (illustrated in Figure 1): *Step 1:* identify themes from both Document 1 (themes from literature) and Document 2 (themes from experiences of psychologist, psychometrist, and test expert participants); *Step 2:* combine overlapping themes of Documents 1 and 2; *Step 3:* place themes in a logical order to form a draft strategic guideline; *Step 4:* analyze themes by applying Clarke and Braun’s (2013) qualitative thematic data analysis approach and analyze data interpretively thorough reading, coding and theme development; *Step 5:* finalize the strategic guideline by ensuring that it includes the elements of transparency, clarity, comprehension and transferability to various psychological assessment contexts.

**Figure 2:**
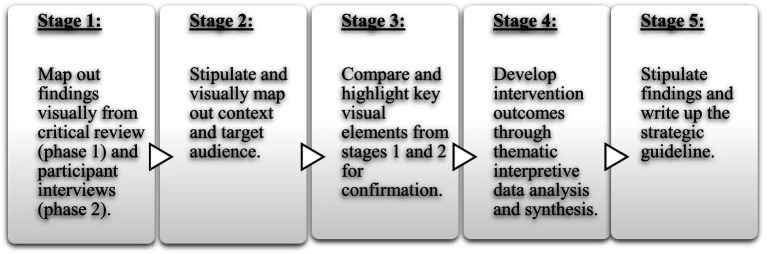
Five-staged method followed for guideline development.

The five-step procedure contained a five-staged method which was followed when developing the strategic guideline, as illustrated in Figure 2. This method was developed from the guideline development methods followed by the World Health Organization [World Health Organization (WHO), 2014]. The five-staged method followed to develop the strategic guideline included (1) *stage 1* and (2) *stage 2*, where the findings of phases 1 and 2 as well as the context and target audience were mapped out on a visual display. Then (3) *stage 3* commenced, where the elements mapped out in *stage 1* (reviewed literature and participant experiences) were highlighted after weighing them up against the context and audience targeted by the interventions recommended in the strategic guideline (elements of *stage 2*). Highlighted elements allowed for confirmation. (4) *Stage 4* entailed thematic interpretive data analysis and synthesis where the highlighted elements were considered critically and interpretively to identify and create new findings that could serve as intervention outcomes. PICO-questions [World Health Organization (WHO), 2014] were additionally asked during this stage, namely questions regarding the *Population* (who is targeted by the recommended intervention), *Intervention* (the action being considered), *Comparator* (any alternative choices of action), and *Outcomes* (considering the purpose of the recommendation). (5) *Stage 5* entailed writing up all the findings in the form of a strategic guideline.

## Findings and discussion

Four main themes with subthemes emerged from the documents analyzed (refer to Table 1). These identified themes served as baseline from which the strategic guideline was developed.

**Table 1:** Themes identified after analysis of the two documents.

Theme number	Theme description
*Theme 1:*	*Utilized intelligence measurements in current South African school learner context are less relevant*
Subthemes:	1.1 Outdated 1.2 Demographically less relevant 1.3 Contextually less relevant
*Theme 2:*	*It does not seem feasible to design or adapt suitable intelligence measures that are valid and reliable in the current South African school learner context*
Subthemes:	2.1 Translation challenges 2.2 Norming and standardisation challenges
*Theme 3:*	*The South African education system is a major issue specifically within lower SES contexts*
*Theme 4:*	*Current South African school learner contextual and demographic influences need to be taken into consideration*
Subthemes:	4.1 Needed input from diverse groups represented in ZA 4.2 Optimal standardisation and updating of measurements 4.3 Selection of applicable instruments 4.4 Constructive interpretation of test performance and consequent diagnosis and intervention plans 4.5 Optimal manner in which intelligence assessment is conducted

### Theme 1: utilized intelligence measurements in current South African school learner context are less relevant

Literature in Document 1 (Van der Merwe et al., 2022) and participants’ experiences in Document 2 (Van der Merwe et al., 2024) revealed that administered intelligence test instruments were unsuitable to the present, multi-ethnic South African school learners, when considering their unique and modern contexts. The lower relevance of intelligence measurements could be ascribed to the available instruments not aligning with the demographics and modern contexts of school learners in ZA. Administration of internationally and locally (many outdated) developed intelligence tests have yielded unequal test performances from multi-ethnic school learners because instruments were developed by experts with differing cultures and contexts, normed on dissimilar populations (with unrelated cultures, languages, contexts, and sometimes time frames), making these intellectual test instruments inapplicable (Cockcroft and Sumaya, 2022; Dutt et al., 2022; Laher and Cockcroft, 2017; Shuttleworth-Edwards, 2023).

As the largest psychometric test developer and supplier, the HSRC has helped to develop the groundwork for South African local psychological testing today (Laher and Cockcroft, 2014; Meiring, 2007). After the HSRC closed their test development research department, there had been constructive research efforts in the field of psychometric measurement development and standardisation, but on a much smaller scale, adding to the shortage of cross-culturally suitable, up-to-date neuropsychological tests (Cockcroft and Sumaya, 2022; Foxcroft, 2004; Laher and Cockcroft, 2014; Lucas, 2013; Shuttleworth-Edwards and Truter, 2023; Shuttleworth-Edwards et al., 2013).

#### Outdated

Participants in Document 2 disclosed that, because the HSRC discontinued large-scale research projects within schools in ZA, utilized intelligence instruments have become outdated (with outdated test items, graphics, language use, and norms) and inapplicable, especially in view of the present South African school learner’s modern world of living. One participant highlighted the historic trend of segregation in ZA and argued that if the psychology departments of different universities, that function as separate entities, had only collaborated forces, then ZA would have been able to develop sufficient intelligence instruments by now. In Document 2 it was also mentioned that even though outdated research has caused many commonly employed intelligence measurements to become irrelevant, the tests still remain valid, as the researched theoretical base has not disappeared. According to the literature in Document 1, mention was made of intelligence assessment instruments being valid and reliable because they retained sound psychometric properties; however, needed to be updated, adapted, and re-normed to appropriately fit the local school learner population being measured. Authors in Document 1 furthermore noted the essential need to update norms of imported intelligence instruments. Shuttleworth-Edwards (2023) and Cockcroft and Sumaya (2022) concurred, stating that administered neuropsychological measurements need to be standardised with locally appropriate norms that are contextually and culturally representative of the current South African population being tested.

#### Demographically less relevant

Performance disparities could be found across different cultural groups as certain test items might hold different meanings for various cultural groups and/or the school learners may not be tested in their primary language (Shuttleworth-Edwards, 2023). In ZA, there are twenty-eight languages spoken (with 12 officially recognized languages) and many of the country’s school learners are multilingual, with not all equally proficient in the language of testing, which is mainly English (Cockcroft and Sumaya, 2022). With few existing neuropsychological tests in indigenous languages, practitioners often need to employ practices such as translating test instruments, providing additional testing time, or appointing translators (where professional translators are not always available) while assessing (Cockcroft and Sumaya, 2022). According to literature critically reviewed in Document 1 and participant comments in Document 2, unfair intelligence assessment practice was noted when considering the multilingual and multicultural formation of the local school learner population group. Both documents viewed these locally administered intelligence tests as unsuitable to South African school learners as measurements were developed and normed on other, differing populations and contexts; school learners’ performance on tests could be hindered due to elements such as unfamiliar language or graphics found in tests, cultural differences in approaching timed tests, and more. In a research study exploring the ethical challenges experienced by psychologists who assessed non-Caucasian school children in ZA, dilemmas identified included “language, cultural knowledge, test-wiseness, confidentiality and communication with parents” (Bayi, 2010, p. 64). Shuttleworth-Edwards (2023) mentions how diagnostic and intervention applications could be inaccurate when administering Western-developed and normed cognitive tests, as they differ from the non-Westernised cultures found in ZA. The inapplicability of Western-developed tests holds the concern of being loaded with the cultural bias due to influencing elements of the test developers (including their understanding and view of the construct of intelligence) and the population used when norming the tests (Bayi, 2010). Dutt et al. (2022) are of the opinion that neuropsychological tests that are developed and normed on Western, mono-cultural, well-educated, English-speaking population groups are inapplicable and should not be administered to non-Western, multicultural and multilingual groups.

#### Contextually less relevant

Both documents mentioned how school learners’ performance on administered intelligence instruments could be affected negatively by unfamiliar test language and content (such as certain graphics or scenarios) that do not reflect the school learners’ current world of living. Additional remarks in both documents stated how differences in the school learners’ contexts (including educational, social, and cultural backgrounds) and thus variances in exposure to language and information would yield differing performances, advising careful interpretation of test scores. Test unsuitability was also noted by Shuttleworth-Edwards (2023), who explained how cognitive assessment is invalid when disparities occur among the various contexts in which South Africans reside, varying from the non-Western impoverished areas to more Western-developed advantaged areas. Administering measurements developed and normed in Western contexts to persons from a disadvantaged, non-Western background (with poorly resourced, lower quality of schooling), would be unfair as their test performances would be hindered by lower levels of literacy and test-taking sophistication, as well as unfamiliarity to test content and language use (Bayi, 2010; Dutt et al., 2022; Shuttleworth-Edwards, 2023).

In view of the above-mentioned statements, it appears improbable for intelligence instruments to be standardised fairly in order to appropriately suite school learners in ZA.

### Theme 2: it does not seem feasible to design or adapt suitable intelligence measures that are valid and reliable in the current South African school learner context

Developing an intelligence test that is applicable, valid, and reliable for South African school learners seems unrealistic, especially given their multilingual and multicultural backgrounds, along with diverse educational environments, which introduces various challenges.

#### Translation challenges

Intellectual assessment in South Africa has the significant challenge of the country having all its multiple races, cultures, and languages scattered among contexts varying in quality of education and level of socio-economic stance (Shuttleworth-Edwards, 2023). It was evident from Documents 1 and 2 that it might be impossible to standardise intelligence measures for school learners in ZA, especially when considering their multidiverse contextual backgrounds. Both documents noted challenges to standardising intelligence measures for school learners with multiple languages and dialects (which include test translation and norming difficulties) and differing levels in quality of education. Influencing elements of differing quality of education, language and culture would most likely result in test performances that do not reflect the testee’s actual ability, causing incorrect diagnoses and suggested interventions (Dutt et al., 2022; Laher and Cockcroft, 2017). Dutt et al. (2022) raise difficult questions regarding influencing contextual and cultural elements that may result in biased assessment outcomes, as well as query assessment validity if practitioners were to deviate from the norm when administrating tests.

#### Norming and standardisation challenges

Some participants in Document 2 questioned the probability of collecting representative norm samples which require excessive resources, especially after the government-supported HSRC was transformed and discontinued large-scale research projects within school environments across the whole of ZA. Since the commencement of the post-apartheid government, the HSRC has lost its government funding and underwent drastic changes in staff, management, relationships with government (specifically the DoE) and universities, as well as its research aims and activities (Chisholm and Morrow, 2007). According to Lucas (2013), test development and adaptation has declined drastically due to large costs and the HSRC changing its roles, such as discontinuing the development of local neuropsychological tests.

### Theme 3: the South African education system is a major issue specifically within lower SES contexts

Document 1 reported on research findings of a downward trend in performance on intelligence tests related to lower quality of education received at schools, regardless of the school learners’ ethnicity and, as stated in one study, irrespective of their home language (see Van Wyhe, 2012). Document 2 concurred by observing substandard education provided at schools located within lower SES environments which hindered the learners’ abilities to perform on intelligence tests. Dutt et al. (2022) mention how higher cognitive skills (which are required to perform in intelligence tests) are attained within contexts of learning such as schools, where the quality and type of educational exposure would predict intellectual test performance. Intelligence tests administered to school learners from a disadvantaged educational background need to be adapted adequately while being weary of over or under-exaggerated scores when interpreting and reporting on findings (Shuttleworth-Edwards, 2023). Western-developed and normed tests like Wechsler intelligence tests have been found suitable to school learners from advantaged educational backgrounds, irrespective of their race, with norms from these school learners being very similar to the American-developed norms (Shuttleworth-Edwards, 2023).

### Theme 4: current South African school learner contextual and demographic influences need to be taken into consideration

It was evident from both documents that when selecting, adapting, or developing intelligence instruments for school learners in ZA, both their demographic elements and environmental backgrounds need to be considered to enable fair assessment practices.

#### Input needed from diverse groups represented in ZA

Foxcroft (2004) suggests that the development/adaptation of a psychological measurement for multi-ethnic testees in ZA should be conducted by a multidiverse team with representatives of all language and cultural groups. The International Test Commission (International Test Commission (ITC), 2019) concurred with this notion, stating that developers, adapters, as well as reviewers of tests for linguistically and culturally diverse populations need to be knowledgeable and skillful in the target languages and cultures. Having diversity in a team has proven to yield benefits of elevated performance, innovation, problem-solving ability, and productivity by tapping into a larger talent pool (Servaes et al., 2022). Participants in Document 2 suggested assembling a team comprising representatives from all the varying cultural and language groups to design an intelligence measurement instrument which is suitable to the multilingual, multicultural school learners of ZA; to do this, the term ‘intelligence’ should first be defined as novel construct within the unique multidimensional context of South African school learners and then be operationalised into a measuring instrument. Document 2 additionally created awareness of obstacles stakeholders have experienced when employing this process, attributing it to their difficulties to move past former negative experiences in this regard. Participants in Document 2, however, remained positive and suggested attempting this project with tolerance and acceptance; joining of forces between various research institutions in ZA was also suggested.

#### Optimal standardisation and updating of measurements

Despite the shortage of culturally relevant intelligence tests in ZA, academics have standardised some neuropsychological measurements for local use that would allow performances that reflect school learners’ abilities more accurately. According to Shuttleworth-Edwards (2023), research has proven Western-developed intelligence tests suitable to Westernised South Africans. Although ZA has the challenge of limited resources, it is imperative that intelligence test instruments administered to non-Westernised individuals be adapted and standardised appropriately (Cockcroft and Sumaya, 2022; Shuttleworth-Edwards, 2023). Cockcroft and Sumaya (2022) advise following the cross-culturally fair assessment guidelines provided by the International Test Commission when standardising and adapting neuropsychological instruments. Documents 1 and 2 also recommend that administered imported and local measurements need to be standardised with updated and adequate norms, language use, and test items that reflect the South African school learners’ demographics, quality of education, and modern, diverse backgrounds to be valid and reliable; this would require continuous normative and measurement adaptation studies on all learner subgroups across ZA.

Al-Jawahiri and Nielsen (2021), as cited in Dutt et al. (2022) have found higher performances from multilingual, multicultural persons who had higher levels of acculturation, and recommended developing appropriate norms that consider acculturation variables. As quality of education has been found to have one of the highest impacts on neuropsychological test performance, it is suggested to adapt well-researched, commonly used cognitive tests according to demographically relevant within-group norms (and not population-group norms) that are stratified according to quality of education (Fernández, 2022; Shuttleworth-Edwards, 2023).

#### Selection of applicable instruments

Both documents advised considering the school learner’s background, including their environment, demographics, type of schooling, and test-sophistication before choosing intelligence measurements to administer; the instrument should accommodate and represent these elements to enable fair, unbiased assessment practices. Both documents specifically brought awareness to cultural differences in approaching timed tests, where many African cultures would rather act responsibly (taking their time) than swiftly and how this notion should be considered during the selection of test instruments and subtests. Dutt et al. (2022) mention cross-cultural differences when performing test tasks of processing speed due to differing views regarding the construct of time and speed, tasks of digit span and verbal fluency that are related to linguistic properties (differing syllabic length of words) and differing cognitive processing styles (holistic, with attention to context vs. analytical, excluding contextual information). Dutt et al. (2022) suggest practitioners demonstrating so-called “cultural competence,” by taking cognizance of the testee’s ethnic background to select applicable tests, score and interpret them appropriately, and communicate findings effectively.

Document 1 reported research findings on translated versions of local intelligence instrument, the Junior South African Individual Scales (JSAIS), having sound psychometric properties; however, in need of updated, applicable norms and test items (familiar or related to the learners’ demographics, world of living, and educational background) to be suitable and fair to the school learner in ZA. There was additional mention in Document 1 of the locally developed Learning Potential Computerised Adaptive Test (LPCAT) being standardised and validated using multicultural samples from multidiverse contexts. JSAIS and LPCAT are among very few intelligence tests designed within the context of ZA. Regarding international intelligence tests, Document 1 noted research findings of the Kaufman cognitive measurements to be valid, reliable, and suitable to the school learners of ZA. After comprehensive intelligence studies of determining correlations among learners’ abilities in cognition, neuropsychological processing, and academic tasks, the Kaufman test batteries were based upon the dual-theoretical foundation of Cattell-Horn-Carroll (CHC) psychometric theory of cognitive abilities and Luria’s neurological theory of processing, which lead to the instrument having validity, reliability, and cross-cultural applicability (Kaufman et al., 2018). Based on the intelligence theory, the Wechsler batteries are the leading cognitive ability measures in the world and of the most frequently administered tests by South African psychologists (Cassoojee, 2020). Comparative analysis of the Kaufman Assessment Battery for Children, Second Edition (KABC-II; Kaufman and Kaufman, 2004) and Wechsler Intelligence Scale for Children, Fifth Edition (WISC-V; Wechsler, 2014) on school learners in rural contexts in ZA, have proven high construct validity and reliability, indicating optimal suitability to local, multi-ethnic school learner populations from lower SES backgrounds (Cassoojee, 2020; Mitchell et al., 2018). For construct validity in psychological measuring instruments, Stone (2021) recommends ensuring that the measurement’s epistemic goals fit its underlying theory. The goal of intelligence measurement should be to yield findings that reflect true performances, upon which appropriate diagnoses and interventions could be drawn (Cockcroft and Sumaya, 2022; Edwards and Young, 2013; Shuttleworth-Edwards, 2023).

#### Constructive interpretation of test performance and consequent diagnosis and intervention plans

Document 1 and 2 commented on varied test performance results due to environmental, demographic, and scholastic background differences. Both documents mentioned how, for this reason, interpretation of results and consequent diagnoses and intervention plans need to be carried out sensibly, especially when a school learner comes from a school within a lower socio-economic context with lower quality of education or when the administered measurement had cultural or linguistic biased elements. When measuring the intelligence of school learners from a disadvantaged SES and educational background, caution should be taken when interpreting and reporting on findings, as biases that form within lower SES contexts (i.e., lower quality of education, poor parental education, poor nutrition and health care, or exposure to violence) would have a negative, unfair effect on learners’ performance (Cockcroft and Sumaya, 2022; Mitchell et al., 2018; Shuttleworth-Edwards, 2023). There is a need to carefully interpret scores for diagnoses and referrals after assessing multicultural and multilingual learners using intelligence tests that are not culturally fair or suitably standardised, as findings are likely not to reflect the school learner’s true ability (Cockcroft and Sumaya, 2022).

#### Optimal manner and context in which intelligence assessment is conducted

Administering intelligence assessment to multi-ethnic school learners often brings about the challenging debate between choosing appropriate response to diversity (that could compromise test validity) versus adhering to the standardised testing principles set to ensure test validity (Edwards and Young, 2013; Laher and Cockcroft, 2017). Edwards and Young (2013) suggest creating optimal assessment contexts and practices when working with multi-ethnic populations from differing contexts, by adding the principals of flexibility and qualitative investigation that are responsive and sensitive to the testee’s unique features and multicultural background. Participants in Document 2 have mentioned how practitioners need to conduct positive and constructive assessment contexts and practices, not only during but also before (preparation) and after the assessment process.

Some participants in Document 2 mentioned laying the groundwork through discussions and networking with relevant parties of the community, inquiring any influencing challenges (e.g., being hungry, travelling far, family difficulties, etc.), preparing school learners to be assessed, as well as managing any power imbalances that may occur to ensure fair practices. Broesch et al. (2020) advise inclusion of the local community to participate in essential stages of the assessment project, especially during the process of preparation. In view of the history of racial tensions in ZA, Cockcroft and Sumaya (2022) recommend incorporating practices that prove consideration of any power relations between the administrator and testee to yield optimal responses and performances in neuropsychological measurements.

### Proposed strategic guideline to design an intelligence instrument applicable to South African school learners

At the beginning of post-apartheid ZA, South African psychologists, psychometrists, and test developers/adaptors realised the need and obligation for psychometric testing that is valid and fair to all (see Foxcroft, 1997, 2004, 2011; Foxcroft et al., 2004; Lucas, 2013). During this time, Foxcroft (1997) published an article which provided essential elements to be considered by a mixed panel of experts during test design and planning for multicultural assessment, namely (a) test purpose and rationale, (b) implications of test design related to testee characteristics, (c) intelligence defined and operationalised from a cross-cultural perspective, (d) framework guiding content development and test specifications, (e) test format, presentation, and response modes, and (f) administration and scoring methods. Many of these features, as well as the above-mentioned themes, have been reflected and built upon to develop the following strategic guideline to design applicable intelligence instruments for South African school learners.

**Figure 3:**
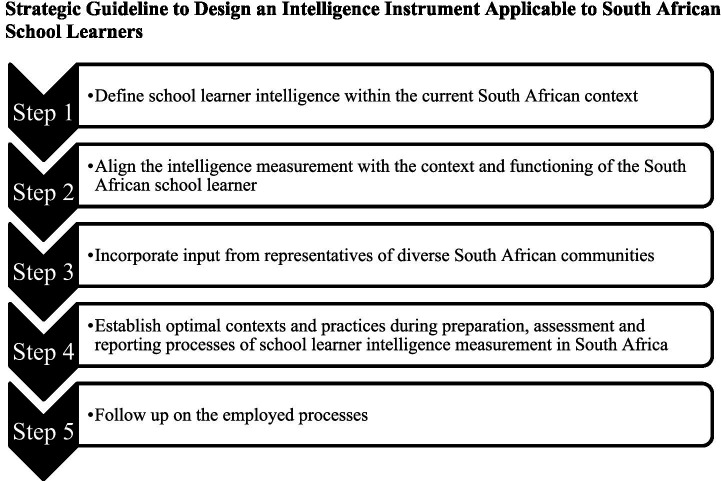
Steps of strategic guideline as identified from themes.

The strategic guideline, as illustrated in Figure 3, consists of five steps.

#### Step 1: define school learner intelligence within the current South African context

The first strategic guideline step suggests defining the concept intelligence of South African school learners within their present contexts. Development of an intelligence measuring instrument should commence by defining the construct being measured (namely intelligence) and then establishing construct validity, namely determining whether the instrument measures the construct it intends to measure (Stone, 2021). This definition would then form part of the underlying theoretical framework, in combination with relevant theories adopted from other intelligence tests, which indirectly inform the designing and validation of intelligence instruments (Stone, 2021). With various existing conceptualizations of intelligence, the constructs of fluid and crystallized intelligence, as proposed by Cattell (1967), have been found most relevant when referring to multi-ethnic, school-going learners who are still undergoing developmental and cognitive changes and residing in differing contexts (Kaufman and Kaufman, 2018; Simpson-Kent et al., 2020). The KABC-II NU (Kaufman and Kaufman, 2018) measuring instrument presents as a good example, being grounded in the dual-theoretical foundation of the CHC and Luria’s theory to provide assessors the flexibility to choose between measuring crystalised and fluid intelligence of scholastic learners, depending on their contextual background; these theories are based upon intelligence research of determining correlations among learners’ abilities in cognition, neuropsychological processing, and academic tasks (Kaufman et al., 2018). It is imperative to conceptualize the construct of intelligence within the relative cultural context; note that intelligence is understood differently in Western cultures (an abstract concept with focus on cognitive capabilities) than in African cultures (emphasis on social behaviour that advances the collective) (Bayi, 2010).

#### Step 2: align the intelligence measurement with the context and functioning of the South African school learner

Step 2 can be followed by applying the PICO model (Richardson et al., 1995) in the manner as described by World Health Organization (WHO) (2014). Firstly, *Population* describes South African school learners according to their relevant demographic characteristics of age, gender, language, health, culture, and other contextual elements (educational, socio-economic, geographic, and social) that affect this population group [World Health Organization (WHO), 2014]. It also depicts acculturation variables where one would take into account the group’s culture according to their ancestral traditional lifestyles and the modern environment with its influencing social and economic elements (Broesch et al., 2020). Secondly, *Intervention* considers intelligence measurement in terms of its efficacy, construct validity, reliability, fairness, and fit according to the school learner context and functioning, whilst keeping the best interest of each school learner in mind [International Test Commission (ITC), 2019; Stone, 2021; World Health Organization (WHO), 2014]. For fairness, the test items should be familiar and appropriate, and test language used should be clear for both the administrators and testees (International Test Commission (ITC), 2019). Thirdly, *Comparator* compares the newly designed instrument to other intelligence instruments to gather existing information to be integrated and built upon [Stone, 2021; World Health Organization (WHO), 2014]. Lastly, *Outcomes* report on test performance (i.e., interpreting results and suggesting interventions such as school/class placement or diagnosis) which should be done in a just and responsible manner of keeping the school learner context and demographics in mind and questioning the potential benefits, harms, and impact on fairness and equity among subgroups or individuals [Stone, 2021; World Health Organization (WHO), 2014].

#### Step 3: incorporate input from representatives of diverse South African communities

Designing of the intelligence measurement should be done by a diverse team with members representing multiple groups found in ZA. Team representatives should be from various relevant regions and departments who deliver a variety of skills, perspectives, and roles [World Health Organization (WHO), 2014]. A diverse team comprising multi-ethnic members holds benefits of enhanced levels of performance, innovation, representivity, equalization, and counteracts bias (Servaes et al., 2022). Practitioners may unwarily be influenced by their own, ethnic backgrounds when making essential interpretations and decisions, arguing as to why a diverse group of individuals with a variety in ethnicity and contexts is recommended (Laher and Cockcroft, 2017). As mentioned earlier in this paper (see themes), when research teams join forces across various universities, departments, and companies in ZA, it could help bridge the challenge of limited resource supply. When working as a diverse team, Servaes et al. (2022) suggest creating a safe and constructive environment by emphasizing notions of diversity, inclusion, and belonging that will allow members to express their ideas freely. When designing intelligence tests, it is important to note the manner and context in which the instrument will be applied., as this will have an impact on the design of the instrument.

#### Step 4: establish optimal contexts and practices during preparation, assessment, and reporting processes of school learner intelligence measurement in South Africa

When working with multi-ethnic populations, Broesch et al. (2020) recommend conducting ethical and culturally appropriate practices. Interacting with cross-cultural school learners should be grounded on “principles of intercultural tolerance and mutual enrichment” (Dichek et al., 2021, p. 210). Applying transformational leadership (influencing others by inspiring and involving them in the tasks at hand) with cultural intelligence (ability to self-reflect and act with insight when interacting with a differing culture) holds promise of creating optimal cross-cultural interactions and healthy environments (Velarde et al., 2022) before, during, and after intelligence measurement practices.

Including the community to participate in the assessment, specifically during the process of preparation, should yield optimal outcomes (Broesch et al., 2020). Particularly during the preparation stage, adequate time should be allowed to explain and take questions from learners and other stakeholders regarding intelligence measurement, gain context-specific knowledge from community members, and ensure obtaining dynamic informed consent through an active community-level discussion (Broesch et al., 2020). Cross-cultural assessments have a higher risk of failing to establish measurement invariance with varying groups portraying response and performance differences to test content and items in intelligence tests (Bader et al., 2021). Therefore Bader et al. (2021) suggest identifying relevant sources of bias, such as method bias (administration of the measuring instrument), item bias (differing interpretation of item content), or language bias (translated tests or multilingual school learners). Gain insight from members of the above-mentioned diverse team (representatives of particular cultures) on how to interpret and apply the performance and results achieved by school learners from differing cultures and contexts (Broesch et al., 2020).

In order to obtain meaningful and true interpretations from multi-ethnic assessments and draw up appropriate diagnoses, recommendations and intervention plans (e.g., placement in a special needs classroom or school), practitioners need to ensure that they do not impose clinical theories on findings that do not reflect information presented by the testee (Cockcroft and Sumaya, 2022; Edwards and Young, 2013; Laher and Cockcroft, 2017). It is advised that the assessor be attentive and responsive to the testees’ non-verbal behaviors (i.e., speech volume, speed and intensity, posture and mannerisms, clothing, and punctuality) that could be influenced by their cultural, SES, educational and home backgrounds (Cockcroft and Sumaya, 2022; Edwards and Young, 2013; Laher and Cockcroft, 2017). Furthermore, they have to be attentive and sensitive to any issues that may arise from cultural or contextual differences and carefully manage any likely power imbalances between the assessee, assessor, and/or other relevant parties, to allow learners the opportunity for optimal test response and performance (Cockcroft and Sumaya, 2022; Edwards and Young, 2013). Practitioners can utilize resources identified from the testee’s environment and test performance during assessment and intervention planning (Edwards and Young, 2013).

#### Step 5: follow up on the employed processes

The processes of designing and implementing intelligence instruments should be accompanied by continuous monitoring, evaluation, and review which could be conducted by an appointed monitoring and review team [World Health Organization (WHO), 2014]. Each selected stakeholder’s relevancy, manner, and level of involvement in this follow-up process should be determined (Boadu and Ile, 2019). When working with multi-ethnic populations, the participatory monitoring and evaluation (PM&E) approach is proposed for this follow-up process, as it poses the following prospective benefits: (1) active participation of all stakeholders (i.e., professionals, academics, the learners, their guardians, and other relevant members of the community); (2) prospect to manage any power disparities that may exist; and (3) higher probability for successful outcomes (Boadu and Ile, 2019).

The team should appraise newly designed intelligence instruments by aiming towards construct validity to ensure fair testing for all learner population groups (Stone, 2021). This could be attained by following the culture, comprehension, and translation bias (CCT) procedure, as introduced by Bader et al. (2021), when designing, standardising, and reviewing measurement instruments. With the CCT procedure, measurement invariance is aspired by identifying the effects of cultural and language bias in tests and then amending those elements until equivalence is reached across cultural groups (Bader et al., 2021). The CCT procedure could be implemented and appraised via continuous standardisation and review of measurement instruments as well as PM&E feedback received from testees and other relevant stakeholders during and after assessment.

## Implications

Administration of culturally fair and sound intelligence tests to school learners in a heterogeneous South African society with various contexts is challenging; however, imperative. Findings from the qualitative document analysis indicated how intelligence measurements and assessment practices could be made more applicable to South African school learners, provided that their demographics and contexts (particularly educational and SES backgrounds) are considered and incorporated into the processes. This, however, needs to be done without compromising the validity of the instrument; it is recommended that the test be based on a theoretical framework, designed uniquely for South Africans and by South Africans (in the form of a multidiverse team). Other than ensuring a valid, reliable, and optimally designed and/or selected intellectual instrument, the manner and context in which this instrument is applied during assessment practices should be regarded equally important, employing practices that include transformational leadership, employing sensitivity and responsiveness, managing any power imbalances, adding flexibility and qualitative inquiry, and conducting ethically and culturally appropriate practices before, during, and after intelligence assessment.

## Limitations and recommendations

Themes were collected after analyzing two research studies (Documents 1 and 2), from which the strategic guideline was created. This qualitative document analysis was limited due to the smaller yield of articles after literature search (see Van der Merwe et al., 2022) and small number of participants (Van der Merwe et al., 2024). Literature in Document 1 was limited to intelligence measurements administered to South African school learners. Participants included in Document 2 were only professionals allowed to and with experience of administering and/or adapting intelligence tests to school learners in South Africa. Although representative of all nine provinces in ZA, data gathered were only from the experienced viewpoint of a selected, smaller number of psychometrists and psychologists. For richer data, more professionals as well as other relevant role players involved in intelligence assessment of local school learners could be interviewed. It is proposed that when employing the suggested strategic guideline to design applicable intelligence instruments (developed from this study’s qualitative document analysis), it is evaluated to ascertain its efficiency, applicability, validity, and reliability.

## Conclusion

Following the aim of this qualitative document analysis, a five-step strategic guideline was developed which provided information to practitioners, in the field of intelligence assessment, on how to design an intelligence assessment instrument that is applicable to South African school learners. It is imperative to note that the manner and context of implementing the intelligence test instrument is just as important as the design itself. The steps introduced were: (1) defining school learner intelligence in the current South African context; (2) selecting a suitable measurement according to the learner’s context and functioning; (3) receiving input from a multidiverse team; (4) establishing optimal assessment contexts and practices; and (5) follow up on the processes applied. To accomplish fair and valid intelligence assessment to multi-ethnic school learners in ZA, practitioners should follow this guideline, to not only yield ethical practice, but also ensure positive assessment experiences and appropriate intervention plans that would benefit the lives of each and every assessed learner, despite their culture, language, or contextual background.

## Data Availability

The raw data supporting the conclusions of this article will be made available by the authors, without undue reservation.

## Author’s note

This manuscript (developing a strategic guideline) was the third and final phase of IM’s Ph.D. thesis entitled: *Development of a strategic guideline to design an intelligence instrument applicable to South African school learners: A multi-method exploration*.
